# Green olive leaf extract (OLE) provides cytoprotection in renal cells exposed to low doses of cadmium

**DOI:** 10.1371/journal.pone.0214159

**Published:** 2019-03-21

**Authors:** Marianna Ranieri, Annarita Di Mise, Graziana Difonzo, Mariangela Centrone, Maria Venneri, Tommaso Pellegrino, Annamaria Russo, Maria Mastrodonato, Francesco Caponio, Giovanna Valenti, Grazia Tamma

**Affiliations:** 1 Department of Biosciences, Biotechnologies and Biopharmaceutics, University of Bari Aldo Moro, Bari, Italy; 2 Department of Soil, Plant and Food Sciences, University of Bari Aldo Moro, Bari, Italy; 3 Department of Biology, University of Bari Aldo Moro, Bari, Italy; 4 Istituto Nazionale di Biostrutture e Biosistemi (I.N.B.B.), Bari, Italy; 5 Center of Excellence in Comparative Genomics (CEGBA), University of Bari, Bari, Italy; University of PECS Medical School, HUNGARY

## Abstract

Cadmium (Cd) is a heavy and highly toxic metal that contaminates air, food and water. Cadmium accumulates in several organs altering normal functions. The kidney is the major organ at risk of damage from chronic exposure to cadmium as a contaminant in food and water. This study aims to investigate the beneficial effects of OLE in renal collecting duct MCD4 cells exposed to a low dose cadmium (1 μM). In MCD4 cells cadmium caused an increase in ROS production, as well as generation of lipid droplets and reduced cell viability. Moreover, cadmium exposure led to a remarkable increase in the frequency of micronuclei and DNA double-strand breaks, assessed using the alkaline comet assay. In addition, cadmium dramatically altered cell cytoskeleton architecture and caused S-glutathionylation of actin. Notably, all cadmium-induced cellular deregulations were prevented by co-treatment with OLE, possibly due to its antioxidant action and to the presence of bioactive phytocompounds. Indeed, OLE treatment attenuated Cd-induced actin S-glutathionylation, thereby stabilizing actin filaments. Taken together, these observations provide a novel insight into the biological action of OLE in renal cells and support the notion that OLE may serve as a potential adjuvant against cadmium-induced nephrotoxicity.

## Introduction

Olive tree products have been widely used in Mediterranean countries in human diet and in traditional medicine [1]. Olive leaves are discarded during olive oil production. Interestingly, recent studies show that olive tree pruning displays the highest pesticide adsorption and might be potentially applied in sustainable bioremediation systems[2]. Indeed, olive leaves represent a natural source of bioactive phytocompounds and their use has been recommended for food preservation. It has been recently shown that olive leaves extract (OLE) reduces lipid oxidation of baked food[3]. In traditional medicine olive leaf brew has been used to cure several diseases including hypertension and hyperglycemia [4,5]. These potential health benefits are supposed to be due to polyphenols and other bioactive compounds displaying peculiar biological properties at different levels[6–8]. Several studies have revealed that olive polyphenols exert anti-inflammatory and antioxidant actions. Moreover, they play a protective role in cancer as they counteract the DNA damage induced by reactive species[9]. Indeed, polyphenols activate transcription factors such as nuclear factor (erythroid-derived 2)-like 2 (Nrf2) that regulates the expression of antioxidant enzymes providing health benefits[10,11].In vivo data indicate that oral OLE supplement prevents cartilage degeneration by increasing hyaluronan[12]. In Swiss albino mice the aqueous extract of olive leaves prevents diclofenac-induced liver injury[13]. Indeed, OLE administration mitigates cell damage and myocardial infarction in Sprague-Dawley rats exposed to air pollution particulate matter (PM) that contains the unsaturated aldehyde acrolein[14]. Furthermore, hydroxytyrosol, which is highly expressed in OLE, protects myocardial H9c2 cell lines against apoptosis induced by CoCl_2_[15].These findings underscore the potential health benefits of OLE in different systems. We have recently demonstrated that the green extract of olive leaves (OLE), obtained from the local *Coratina* cultivar, displays antioxidant activity in NCI-H292 carcinoma cells isolated from human lung[16]. Here, the effect of this extract was investigated in renal collecting duct cells exposed to a low dose of cadmium (1μM). Cadmium (Cd) is an environmental pollutant that contaminates air, food and water causing several health problems. The degree of Cd-induced cytoxicity depends on dose and duration of exposure. High cadmium doses are >10 μM, whereas low doses of cadmium are concentrations between 1–10 μM[17]. In Human Renal Mesangial Cells (HRMCs), a concentration of 4 μM CdCl_2_ for 24 hours is considered to be a low dose treatment[18].

Cadmium has cytotoxic and genotoxic effects as it promotes DNA strand breaks and micronuclei formation [19].Cadmium is a class I carcinogen displaying adverse effects on many organ systems such as bones, lungs and kidneys[20,21]. The kidney is the major organ at risk of injury from chronic exposure to cadmium [20]. In mesangial cells cadmium exposure leads to a decrease in the rate of actin polymerization and an increase in actin depolymerization[22]. Proper cytoskeletal architecture is crucial to normal morphology and cell physiology. In renal cells, cadmium increases S-glutathionylation of actin in a concentration dependent manner[23]. S-glutathionylation is a post-translational modification which is crucial for cells to translate compartmentalized changes in redox signal molecules[10,24]. S-Glutathionylation of actin at cysteine 374 impairs actin polymerization ability, thereby altering cytoskeleton remodeling and cell spreading [25,26]. Redox regulation of target proteins is recognized as being important under physiological and pathological conditions [27]. Cadmium promotes oxidative stress because it inhibits several redox-sensitive enzymes, such as catalase, in different systems [19,28]. In the kidney, cadmium is highly retained, and its concentration is correlated to that detected in the urine. Chronic exposure to cadmium leads to glomerular and tubular dysfunctions that eventually cause renal failure. Importantly, deregulations of renal physiology associated with high cadmium exposure are well described, whereas the effects of low dose exposure need to be better clarified. The present study was undertaken to investigate whether OLE may exert beneficial effects on cadmium treated cells. To this end several biomarkers of cadmium exposure were applied in renal collecting duct MCD4 cells [29].Cell viability, ROS generation, DNA damage and cytoskeleton dynamics were monitored to evaluate the protective effects of OLE.

## Material and methods

### Chemical and reagents

All chemicals were purchased from Sigma-Aldrich. *tert*-Butyl hydroperoxide (tBHP) was a kind gift from A. Signorile (University of Bari). Actin, Glutathione antibodies (D8) were purchased from Santa Cruz Biotechnologies (DBA, Italy). BioGEE, DMEM, GlutaMAX, and fetal calf serum were purchased from Life Technologies (Monza, Italy).

### Olive leaf extract production and chemical characterization

The olive leaves (*cv*. Coratina) were picked off the trees in an olive grove in the province of Bari (40°51'16.2"N 16°47'23.6"E), stored at 4°C and processed in less than 24 h. After washing with tap water at room temperature, the olive leaves were dried at 120°C for 8 min in a ventilated oven (Argolab, Carpi, Italy) to reach a moisture content <1% and then milled in a blender (Waring-Commercial, Torrington, CT, USA). The extraction from leaves was performed in an ultrasound bath (CEIA, Viciomaggio, Italy) adding water (Eth-0) or hydroalcoholic solutions to30% (Eth-30) and 70% ethanol (Eth-70) in a 1/20 ratio (w/v). Three washes were done, each one for 30 min at 35±5°C. Finally, the extracts were filtered through Whatman (GE Healthcare, Milan, Italy) filter paper (67 g m^-2^), lyophilized and stored at -20°C. The total phenol content, the antioxidant activity and the identification of single phenolic compounds were performed according to Difonzo et al. [16]. The Eth-0 OLE showed a content of polyphenols, determined by Folin-Ciocalteu, equal to 180 mg g^-1^ gallic acid equivalents (GAE) and an antioxidant activity, determined by ABTS (2,2′-azino-bis(3-ethylbenzothiazoline-6-sulfonic acid diammonium salt), accounting for 750 μmol TE (Trolox equivalents) g^-1^. The main phenolic compounds found in OLE were oleuropein, hydroxytyrosol glucoside, luteolin-glucosides, verbascoside, ligstroside, secologanoside and other minor compounds, detected by UHPLC-ESI-MS/MS as described in Difonzo et al. [16]. Cadmium detection was carried out by means of inductively coupled plasma–atomic emission spectrometry (ICP-OES) (IKAP 6500, Thermo Scientific, USA). The detailed ICP-OES analytical conditions were the following: from room temperature to 190°C in 15 minutes followed by 10 min hold at the same temperature. For this purpose, an aliquot of 250 mg of olive leaf extract was digested with 8 mL HNO_3_ (69.0%) and 1 mL H_2_O_2_ (30%) using a microwave digestion system (Discovery-SP, CEM, USA).

### Cell culture and treatments

Mouse cortical collecting duct MCD4 cells derived from M1 cells were stably transfected with the water channel AQP2 [29]. MCD4 cells were cultured as described [29,30]. Briefly, cells were grown in Dulbecco’s Modified Eagle’s Medium (DMEM/F12) supplemented with 5% fetal bovine serum, 2 mM L-glutamine, 100i.u./ml penicillin, 100 μg/ml streptomycin and 5 μM dexamethasone at 37°C, 5% CO_2_. Cells were incubated O/N with OLE at increasing concentrations (0.001mg/ml; 0.01mg/ml; 0.1mg/ml). Alternatively, cells were treated with cadmium (0.1μM O/N) in the absence or presence of OLE (0.01mg/ml).

### Crystal violet assay

Crystal Violet assay was performed as previously shown [31] with some adaptations. MCD4 cells were grown in a 96-well plate. After treatment, MCD4 cells were fixed with 4% paraformaldehyde in phosphate buffer saline (PBS) for 20 minutes. After washing, cells were incubated with a solution containing 0.1% crystal violet in 20%methanol and lysated with 10% acetic acid. Cell viability was detected by measurement of the optical density at 595nm (DO_595_) with a Microplate Reader (BIORAD).

### ROS detection

ROS were detected as previously described [24]. After treatment, MCD4 cells were incubated with dihydrorhodamine-123 (10μM) in PBS for 30 min at 37°C with 5% CO2 and recovered incomplete medium for 30 min. In the last 15 min of recovery, cells were treated with *tert*-Butyl hydroperoxide (tBHP, 2 mM for 30min). Cells were lysed in a buffer containing 1%Triton X-100 150 mM NaCl, 25 mM HEPES (pH 7.4). Lysates were analyzed using anRF-5301PC fluorimeter (excitation wavelength,512 nm; emission wavelength, 530 nm).

### Actin staining

Actin staining was performed as already shown [32]. In brief, MCD4 cells were grown on glass coverslips and fixed with 4% paraformaldehyde in phosphate buffer saline (PBS) for 20 minutes. Cells were washed 3 times for 5 minutes in PBS and permeabilized with 0.1% Triton X-100 in PBS for 10 minutes. The actin cytoskeleton was visualized by incubation with phalloidin TRITC (100 μg/ml, 45minutes). The coverslips were mounted using Mowiol to retard quenching of the fluorescence. Actin cytoskeleton was detected by confocal laser scanning microscopy (Leica TCS SP2 camera; Leica Microsystems).

### Actin polymerization assay

Actin polymerization was analyzed as previously described [32,33]. Briefly, MCD4 cells were treated as described above. The treatments were stopped by adding 450 μl of 3.7% paraformaldehyde, 0.1%Triton X-100, 0.25 μM TRITC-phalloidin in 20 mM potassium phosphate, 10 mM PIPES, 5 mM EGTA and 2 mM MgCl2, pH 6.8. After staining for 1 hour, cells were washed three times with PBS and 800 μl of methanol was added overnight. The fluorescence (540/565 nm) was read in a RF-5301PC fluorimeter. The values obtained were analyzed by One-way ANOVA, followed by Tukey’s Multiple Comparison test.

### Micronuclei

MCD4 cells were treated as described above. After treatment, cells were fixed with 4% paraformaldehyde in phosphate buffer saline (PBS) for 20 minutes. Cells were washed 3 times for 5 minutes in PBS and permeabilized with 0.1% Triton X-100 in PBS for 10 minutes. Nuclei were stained with 4′,6-diamidino-2-phenylindole (DAPI) in PBS for 15 min prior to mounting. Nuclei and individual micronuclei were counted manually from DAPI stained cells. At least 300 cells were counted for each experiment and experiments were repeated 5 times.

### Comet assay

MCD4 cells were seeded onto 60-mm dishes and incubated in humidified 5% CO_2_ incubator at 37 °C. After treatments, cells were washed with PBS, detached from the supports and centrifuged at 1000xg for 5 minutes. Cell pellets were resuspended in 20μl in low melting point agarose (LMA, 1% in phosphate buffer saline PBS) and spread on pre-coated slides (normal melting agarose NMA 1% in PBS) and covered with a coverslip. A third agarose layer (150μl of LMA1% in PBS) was then added. The slides were immersed for 1 hour in freshly prepared ice-cold lysis buffer containing 2.5M NaCl, 0.1 M Na_2_EDTA, 0.01 M Tris, 1% Triton X-100, 10%DMSO, adjusted to pH 10 with NaOH) for 1h. The specimens were then subjected to electrophoresis in a buffer containing 0.3M NaOH, 1mM Na_2_EDTA, pH = 13. The slides were washed twice in a neutralization buffer (0.4 M Tris-HCl, pH 7.5) for 5 minutes and then stained with 100ng/ml DAPI. Quantitative assessment of DNA damage was performed using ImageJ analysis software.

### Oil-red-O staining

MCD4 cells were treated as indicated above. Cells were stained with oil red O solution (0.12%) and counterstained with hematoxylin. The specimens were evaluated under light microscopy (Nikon, Instruments SpA, Calenzano, Italy). For image analysis, at least six digital pictures were randomly taken for each sample and for each experimental condition. Original magnification was set at 60×. The obtained data were analyzed using an Image J–Particles Analyzer. Statistical analysis was performed with GraphPad Prism.

### Pyrene actin polymerization assay

MCD4 cells were grown on Ø150 mm Petri dishes at 80% confluence. After treatment cells were scraped and re-suspended in General Actin buffer containing 5 mM Tris-HCl pH 8.0 and 0.2 mM CaCl_2_and protease inhibitors. Cells were homogenized using a 27-gauge needle. Nuclei were removed by centrifugation at 800 x g for 10 min. Cytosol fractions were obtained by centrifugation at 150.000 x g for 1 h at 4°C in a Beckman Rotor TLA 120.1.

G-actin stock was prepared following the manufacturer’s procedures (Cytoskeleton, Inc, #AP05-A).

Cytosol (200 μg) was added in a cuvette and the fluorescence emission signal was recorded using a fluorimeter (RF-5301PC, Shimadzu Corporation, Kyoto Japan) at excitation and emission wavelengths of 370 and 430 nm, respectively. After 3 min, 100 μl of General Actin Buffer (0.4 mg/ml) was added and the fluorescent signal was acquired for the next 3 min. Formation of F-actin was initiated by a 10-fold Actin Polymerization buffer containing 100 mM Tris-HCl pH 7.5, 500 mM KCl, 20 mM MgCl_2_ and 10 mM ATP and the fluorescent emission signal was recorded for 20 min.

### S-Glutathionylation assay

MCD4 cells were seeded onØ40-mm glass coverslips and grown to 80% confluence. During the last hour of treatment, cells were incubated for 1 h with BioGEE reagent (250μM)at 37°C, 5%CO_2_, as previously described [24]. Cells were lysed in a buffer containing 1% Triton X-100, 150 mM NaCl, and 25mM HEPES (pH 7.4). Lysates were clarified from cellular debris by centrifugation at 13.000 x g for 10 min at 4°C and then incubated with streptavidin-agarose for 5h at 4°C. After three washes in the above-mentioned lysis buffer, glutathionylated proteins were resuspended in 50μl of Laemmli’s buffer, in non-denaturating conditions, and subjected to immunoblotting using anti-actin and anti-glutathione antibodies. Obtained bands were normalized to total protein using the stain-free technology (Bio-Rad, Segrate Milano Italy).

### Statistical analysis

Data are reported as mean ± SEM. Statistical analysis of data was performed with GraphPad Prism. One-way ANOVA, followed by Tukey’s Multiple Comparison test, was applied. P<0.05 was considered statistically different.

## Results

### Characterization of olive leaves extract in renal cells

**Fig 1:**
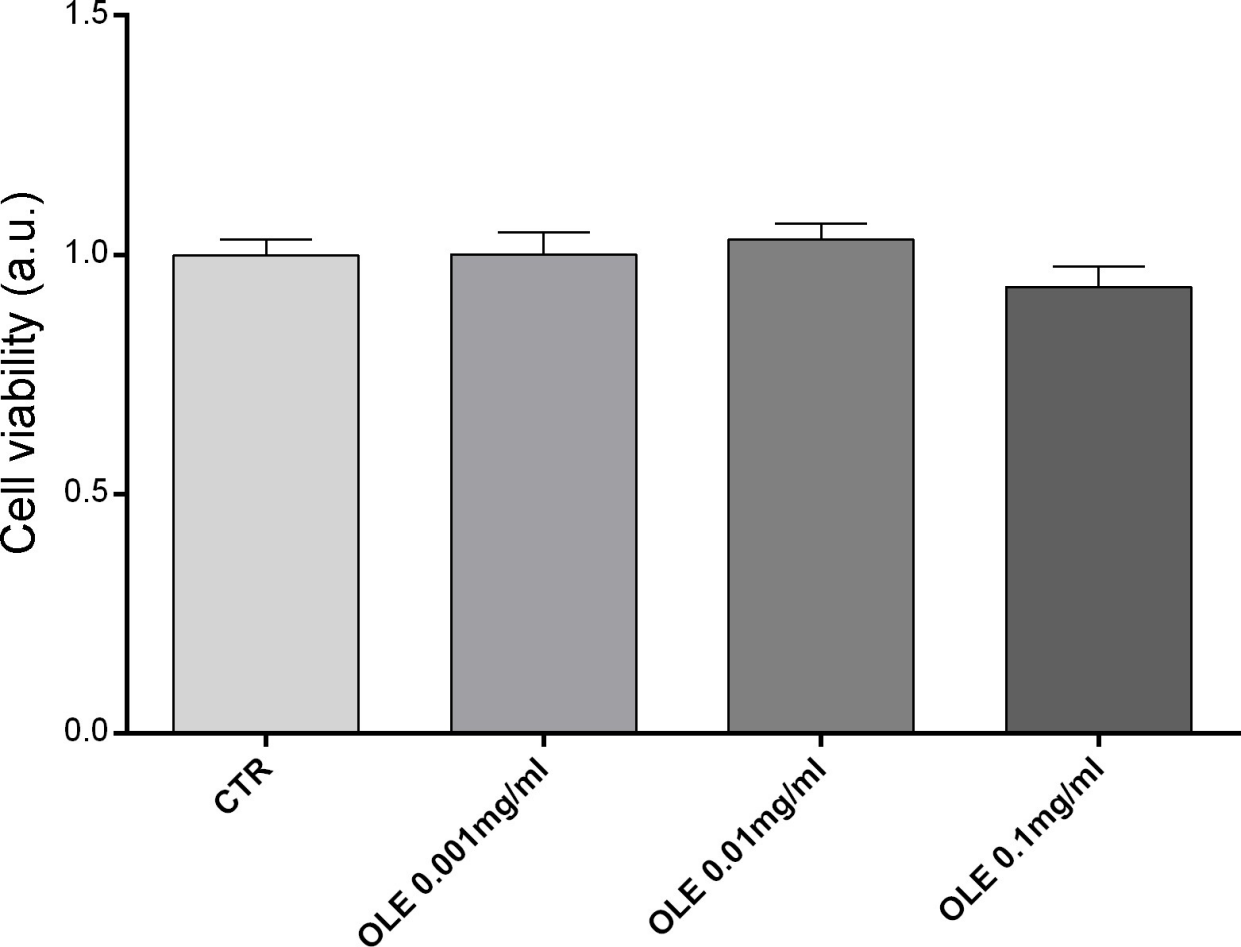
Cell viability of MCD4 cells. Cells were left under basal condition or treated with OLE (0.001mg/ml; 0.01mg/ml; 0.1mg/ml) and were stained with crystal violet solution. Data are presented as means ± SEMs of 3 independent experiments.

Several phytocompounds known for their antioxidant actions may instead promote cytotoxicity by inducing ROS generation [34]. Here, the potential effects of OLE on cell viability were investigated by applying the crystal violet assay (see Methods for details). Cells were incubated O/N with OLE at increasing concentrations (0.001mg/ml; 0.01mg/ml; 0.1mg/ml). Compared to cells left under control condition, OLE treatment does not affect cell viability at the concentrations used (Fig 1).

**Fig 2:**
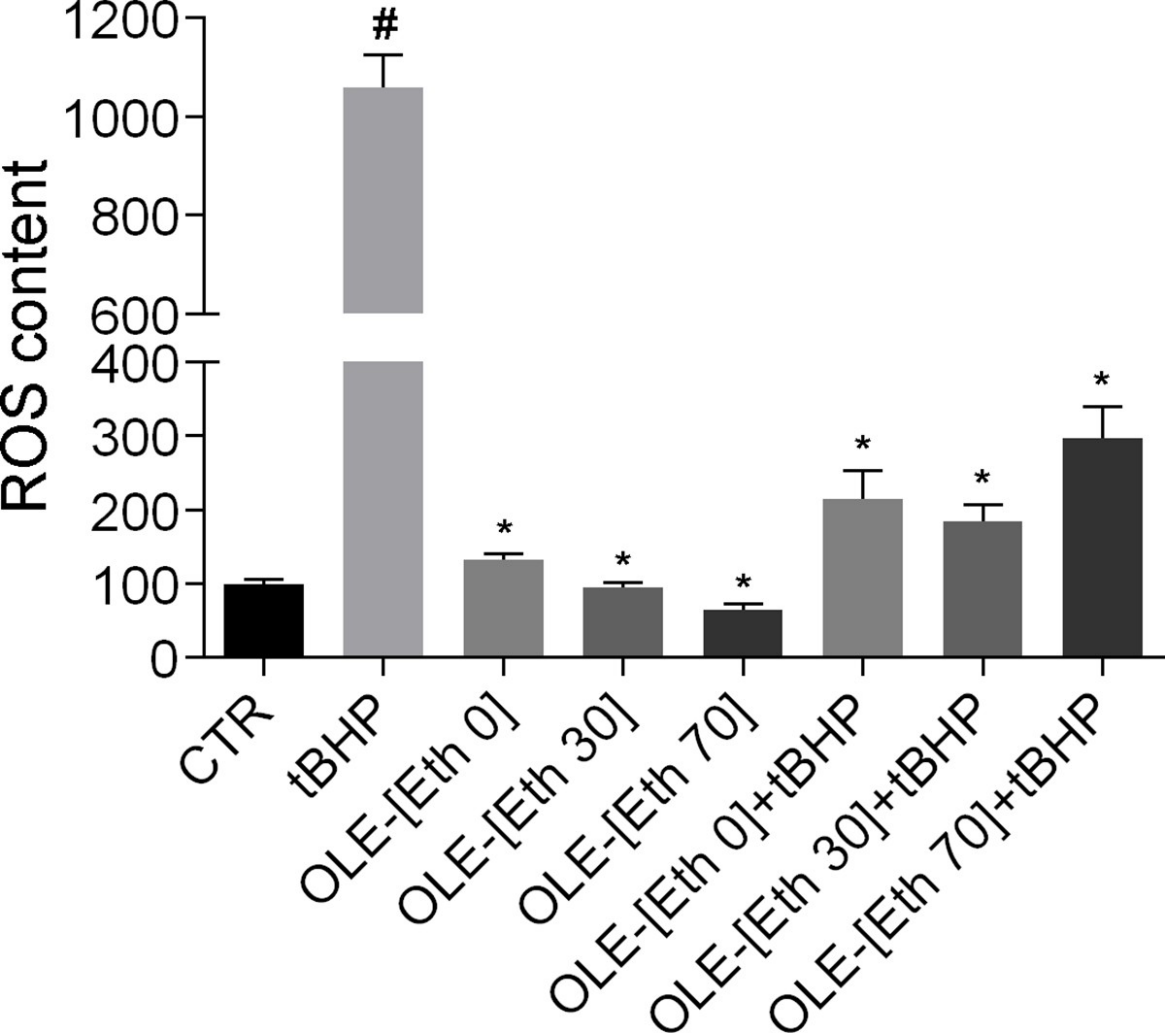
ROS content. ROS content was measured using dihydrorhodamine-123 fluorescence in MCD4 cells treated as described in Methods. As positive control, cells were treated with the oxidant tBHP. Data are shown as mean ± SEMs and analyzed by one-way ANOVA followed by followed by Tukey’s Multiple Comparison test. (#P<0.0001 vs CTR; *P<0.0001 vs tBHP).

Next, the antioxidant effect of OLE (0.01mg/ml) obtained by different green extraction methods [16] was evaluated. Compared to the positive control (cells treated with the oxidant tBHP), cells treated with OLE, obtained with water as extraction solvent (Eth-0), displayed a similar ability to reduce intracellular ROS induced by tBHP as the cells treated with the extract obtained with ethanol (Eth-30 and Eth-70; Fig 2).

**Fig 3:**
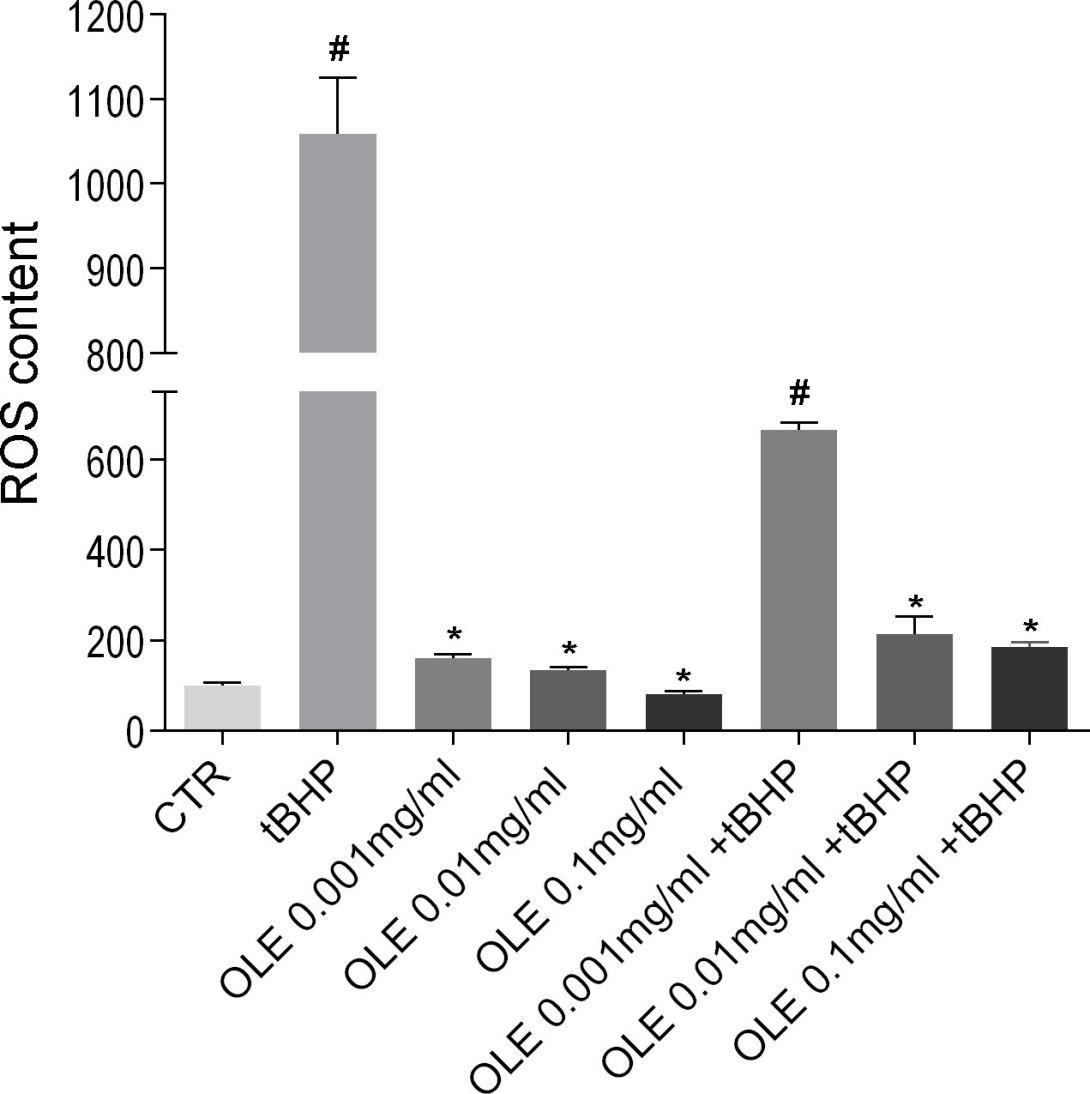
ROS content. ROS content was measured using dihydrorhodamine-123 fluorescence in MCD4 cells treated as described in the Methods section. As positive control, cells were treated with the oxidant tBHP. Data are shown as mean ± SEMs and analyzed by one-way ANOVA followed by followed by Tukey’s Multiple Comparison test. (#P<0.001 vs CTR; *P<0.001 vs tBHP).

Since the extract obtained without ethanol has a similar effect as the extract obtained with ethanol, ROS content was therefore evaluated in MCD4 cells incubated with increasing concentrations of OLE obtained without ethanol (Fig 3).

Compared to the positive control, cells co-treated with the oxidant tBHP and OLE extracts at 0.01mg/ml or 0.1mg/ml concentrations, displayed a similar and significant decrease in ROS generation induced by tBHP (Fig 3).

Incubation with the OLE alone, at increasing concentrations (0.001mg/ml; 0.01mg/ml; 0.1mg/ml), does not alter ROS content. Therefore, OLE was used at 0.01mg/ml for subsequent analysis. These data indicate that OLE (Eth-0) has an antioxidant effect in renal cells, similar to that observed in NCI-H292 lung cells [16].

**Fig 4:**
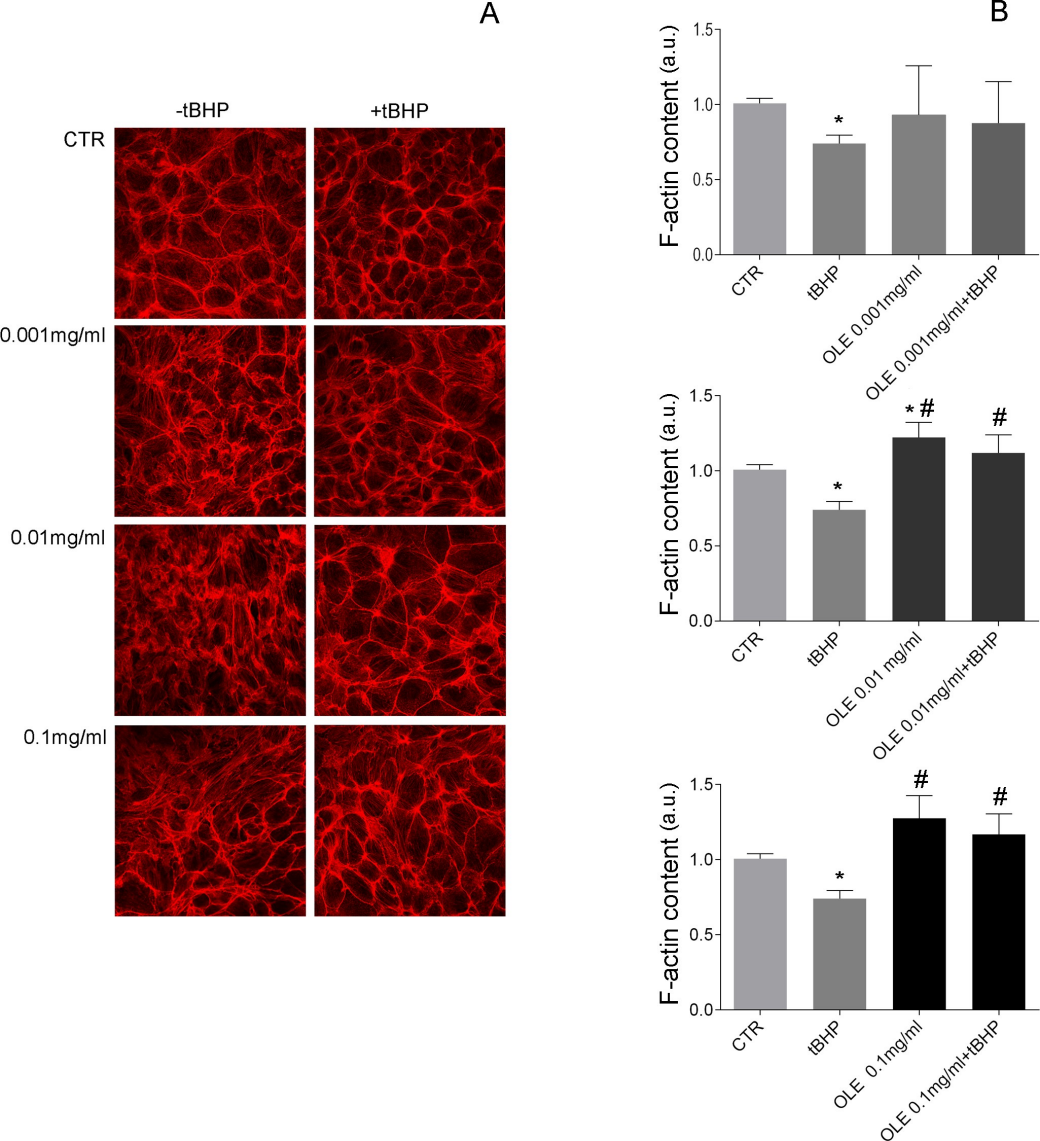
Staining of F-actin in MCD4 cells. (A) Cells were left under basal condition or treated with OLE (0.001mg/ml; 0.01mg/ml; 0.1mg/ml) in the presence or in the absence of the oxidant tBHP (2 mm) for 30 min. Specimens were incubated with Phalloidin-TRITC (400 μg/ml) for 45 min at room temperature to detect F-actin. (B) F-actin content was semi-quantified by actin polymerization assay. Confluent cells were treated as described under methods. After staining with TRITC-phalloidin, cells were extracted with cold methanol and the fluorescence absorbance of extracts was read (540/565 nm) in a RF-5301PC fluorimeter. The values obtained were compared using one-way Anova and Tukey’s multiple comparison test (*P<0.05 vs CTR; #P<0.01 vs tBHP).

ROS signals regulate actin cytoskeleton remodeling via Rho proteins [35,36]. ROS production results in Rac1 activation that causes RhoA inactivation and actin depolymerization [37]. To investigate the effect of OLE on actin cytoskeleton remodeling, increasing concentrations of OLE (0.001mg/ml; 0.01mg/ml; 0.1mg/ml) were applied in MCD4 cells (Fig 4A). The data indicate that OLE stabilizes actin filaments and attenuates actin depolymerization induced by the oxidant tBHP. Semi-quantitative analysis of the amount of F-actin evaluated with the actin polymerization assay (Fig 4B) confirmed that F-actin content significantly decreased on preincubation with tBHP. In contrast, incubation with OLE increased F-actin content and prevented tBHP-induced actin depolymerization.

### Olive leaf extract effects on renal cells exposed to low cadmium

Protective action of antioxidants has been tested in neuroblastoma cells exposed to heavy metals including cadmium [38]. Inductively coupled plasma-atomic emission spectrometry analysis revealed that cadmium was not detected in the olive leaves extracts used in this study. Here, the potential beneficial effects of OLE on cells exposed to a low dose of cadmium was evaluated by applying several assays. Renal collecting duct MCD4 cells were left untreated or incubated with OLE. Alternatively, cells were incubated with cadmium or co-treated with OLE and cadmium.

**Fig 5:**
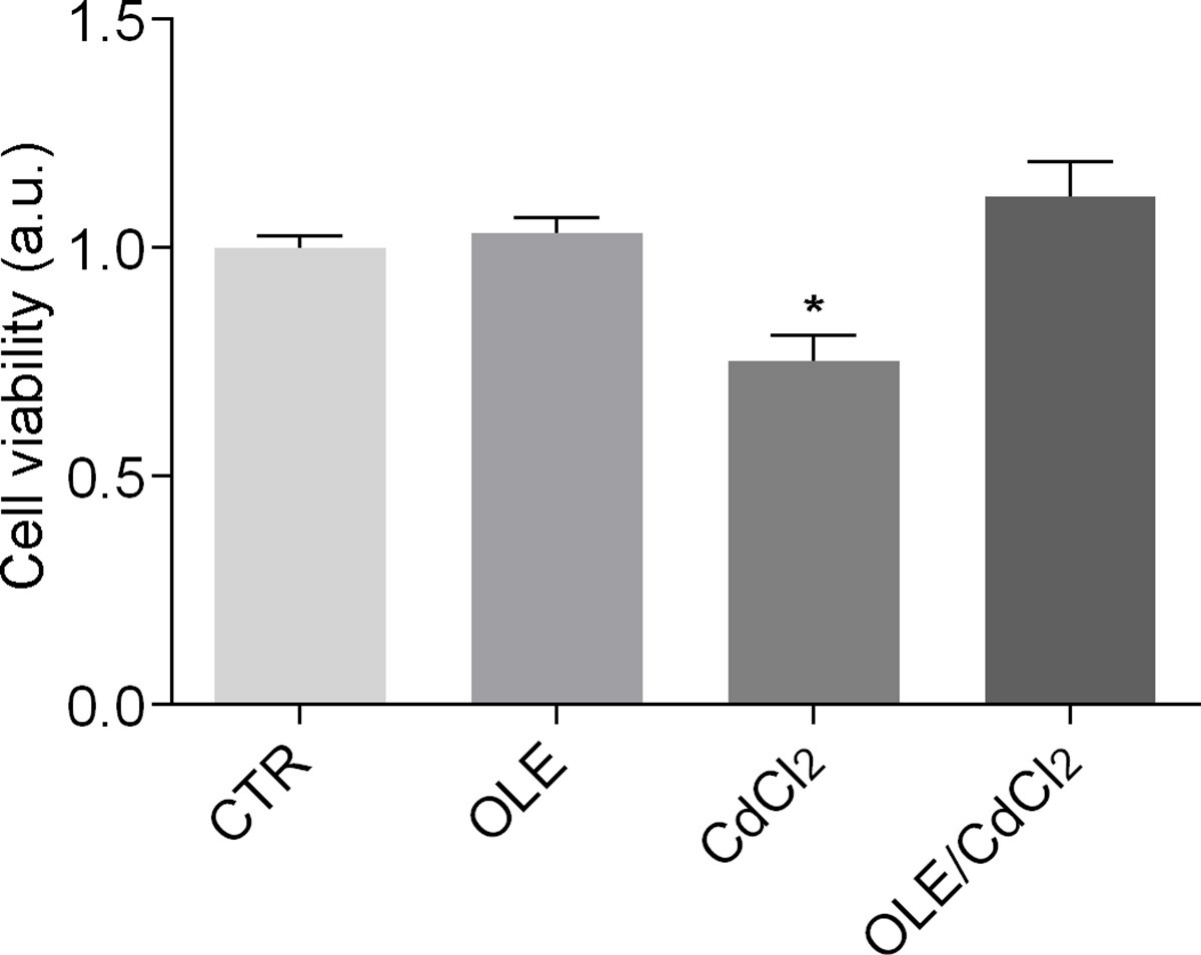
Cell viability of MCD4 cells. Cells were left under basal conditions or treated with OLE (0.01 mg/ml), CdCl_2_ (0.1μM) or with OLE in the presence of CdCl_2_. After treatment, cells were stained with crystal violet solution. Data are presented as means ± SEMs of 3 independent experiments (*P< 0.001 vs CTR).

Crystal violet assay revealed that low cadmium exposure (0.1μM O/N) significantly reduced renal cell viability (Fig 5).

**Fig 6:**
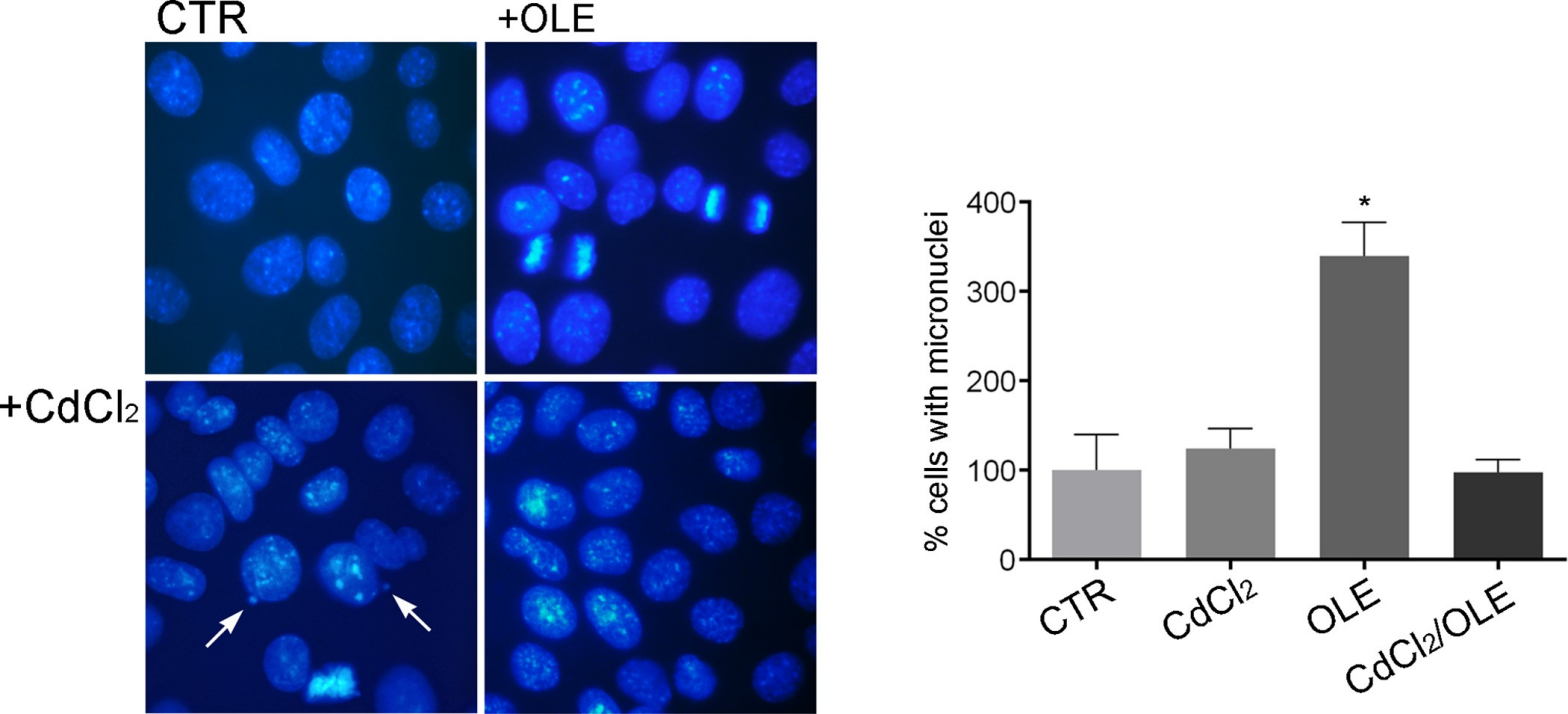
Determination of micronuclei. Nuclei were detected using DAPI staining. Percentage of micronucleated cells was enumerated according to DAPI staining. Cadmium treatment promotes micronuclei formation compared to untreated cells. Data are shown as mean ± SEMs and analyzed by one-way ANOVA followed by followed by Tukey’s Multiple Comparison test. (*P< 0.001 vs CTR).

Interestingly, this toxic effect was prevented by co-treatment with OLE. Moreover, a significant induction of micronuclei formation, a general biomarker to test cell exposure to genotoxic pollutants, was only detected in cells incubated with cadmium alone (Fig 6).

A reduction in micronuclei formation, instead, was measured in cells co-incubated with OLE and cadmium. Incubation with OLE had no relevant effect on micronuclei formation (Fig 6).

**Fig 7:**
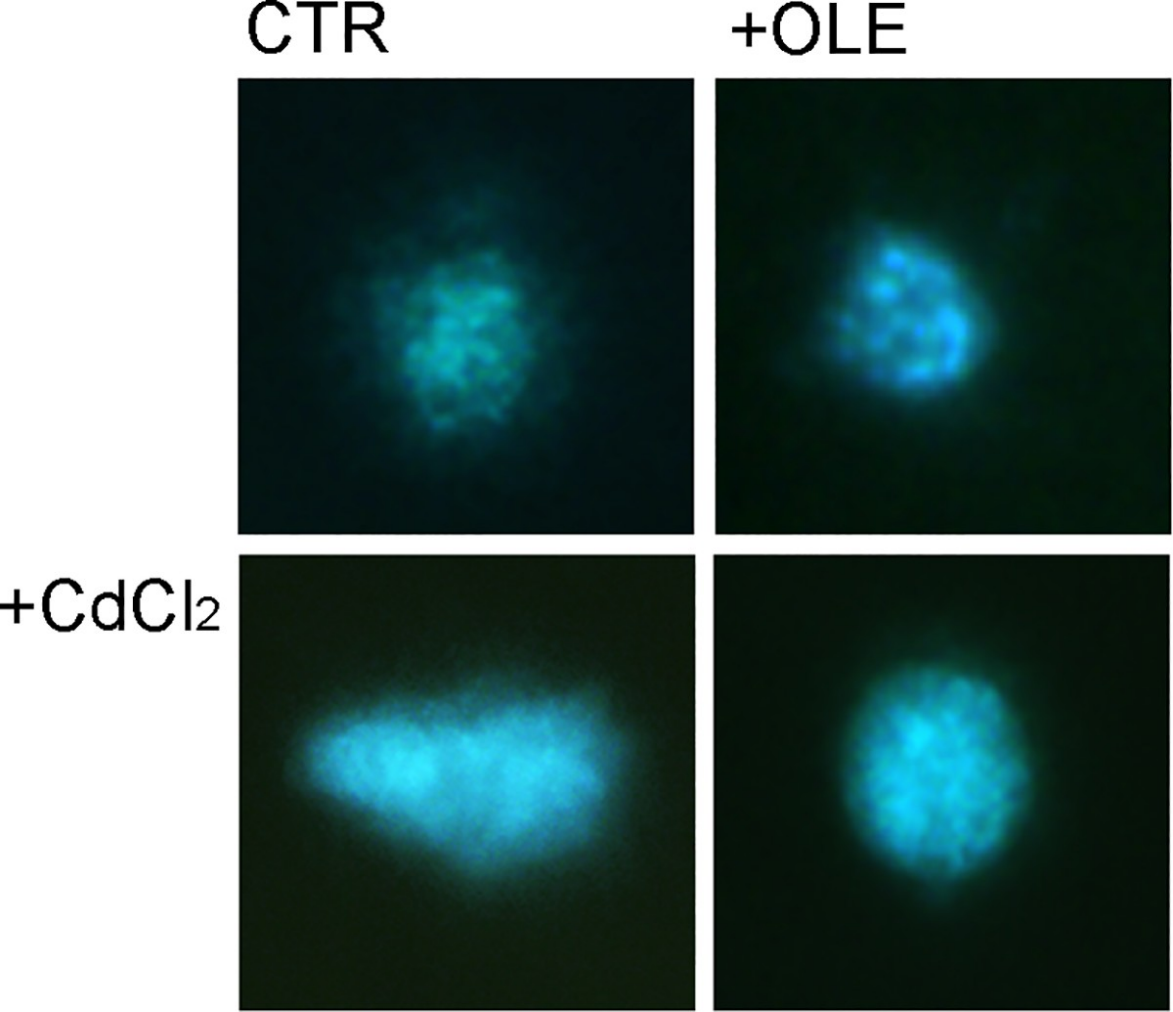
Comet assay. DNA damage was evaluated in MCD4 cells treated with OLE (0.01 mg/ml), CdCl_2_ (0.1μM) or with OLE in the presence of CdCl2. Imaging analysis revealed that cadmium promotes tail formation. This effect was counteracted by co-treatment with OLE.

Strand breaks of DNA were further evaluated using the comet test (Fig 7).

Once imaging was complete, cellular analysis was performed using Image J. The data are summarized in Table 1 showing that treatment with cadmium significantly increased the tail moment, the tail DNA % and the tail length/cell length compared to cells left under control conditions. Co-incubation with OLE and cadmium reduced these parameters compared to those measured in cells treated with cadmium alone.

**Table 1:** Comet parameters were obtained using Image J.

	CTR	OLE	CdCl_2_	OLE/ CdCl_2_
Tail Moment	15122±812.8	15122±1105^#^	111586±11151*	34993±2517*^#^
Tail DNA %	15.28±0.3820	17.30±0.5443^#^	61.74±1.418*	45.71±0.8113*^#^
Tail length/Cell length	0.5199±0.02029	0.5865±0.03525^#^	2.207±0.07510*	0.4522±0.02493*^#^
Cell number	199	80	105	235

The tail moment, the tail DNA %, the tail length/cells are reported here.

*P<0.0001 vs CTR

^#^P<0.0001 vs CdCl_2_

**Fig 8:**
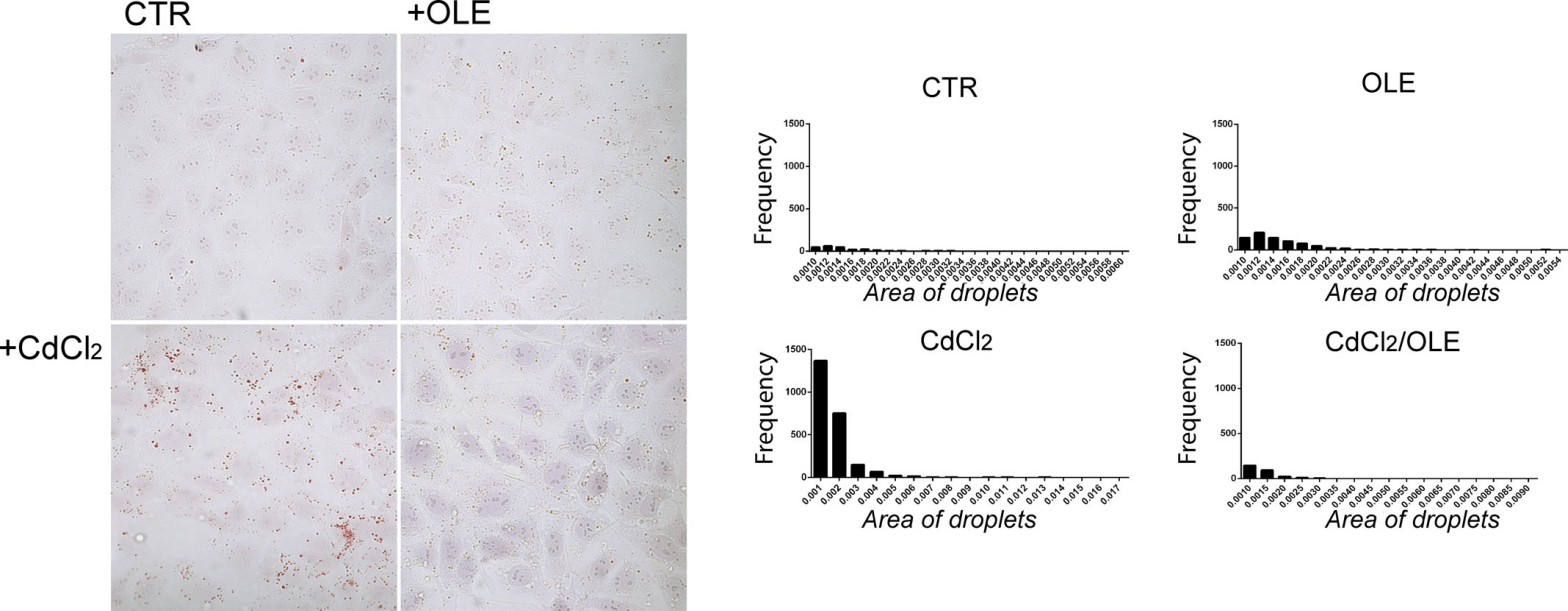
Detection of intracellular lipid droplets. (A) Oil Red O staining for lipid droplet formation, the lipid droplets are stained dark red. (B) The number and the area of lipid droplets are shown by frequency distribution.

Cadmium exposure is known to affect lipid metabolism even at low concentrations [39–41] and to cause a dose dependent increase in plasma triglyceride levels [42]. Using Oil-Red-O staining, which is able to dye neutral triglycerides and glycolipids, we found that cadmium induced lipid droplet accumulation that was not observed when cells were co-treated with OLE and cadmium (Fig 8A).

The data were analyzed using Image J and are summarized in Table 2.

**Table 2:** Lipid droplets analysis was performed with Image J.

	CTR	OLE	CdCl_2_	OLE/ CdCl_2_
% cells with lipid droplets	7.7±1.34	14.96±2.22^#^	66.17±6.62*	13.10±2.34^#^
Droplets/cell	1.9±0.47	3.08±0.57^#^	10.02±1.85*	1.44±0.31^#^
Cell number	766	1194	664	986

The percentage of cells with lipid droplets, droplets/cell and cell number are reported here.

*P<0.0001 vs CTR

^#^P<0.0001 vs CdCl_2_

Treatment with cadmium increases the bulk of cells expressing lipid droplets, the number and the area of lipid droplets as shown by the frequency distribution (Fig 8B). These effects were not observed in cells co-treated with OLE.

**Fig 9:**
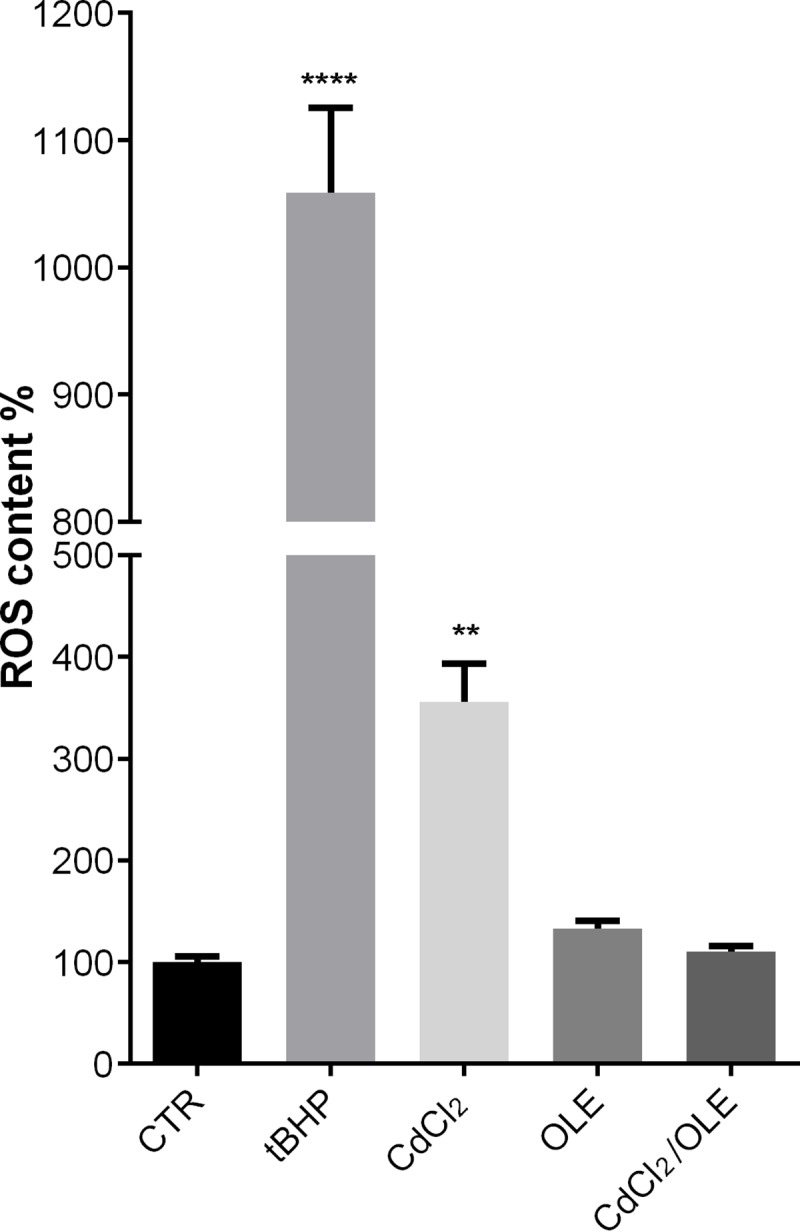
ROS content. ROS content was measured using dihydrorhodamine-123 fluorescence in MCD4 cells treated as described in Methods. As positive control, cells were treated with tBHP. Data are shown as mean ± SEMs and analyzed by one-way ANOVA followed using Tukey’s Multiple Comparison test. (**P<0.01 vs CTR; ****P<0.0001 vs CTR).

Generation of reactive species induced by cadmium exposure may account for the most observed toxic effects. Therefore, we next evaluated whether OLE may be useful to modulate cadmium induced ROS generation. To this end, the fluorescent probe dihydrorhodamine-123 was applied to MCD4 cells after the treatment described above. The data summarized in Fig 9 indicate that OLE significantly reduced the increase in ROS induced by cadmium. Together these findings suggest that OLE may play a beneficial role and stimulate antioxidant actions upon low cadmium exposure.

### Olive leaf extract modulates actin remodeling and glutathionylation on renal cells exposed to low cadmium

**Fig 10:**
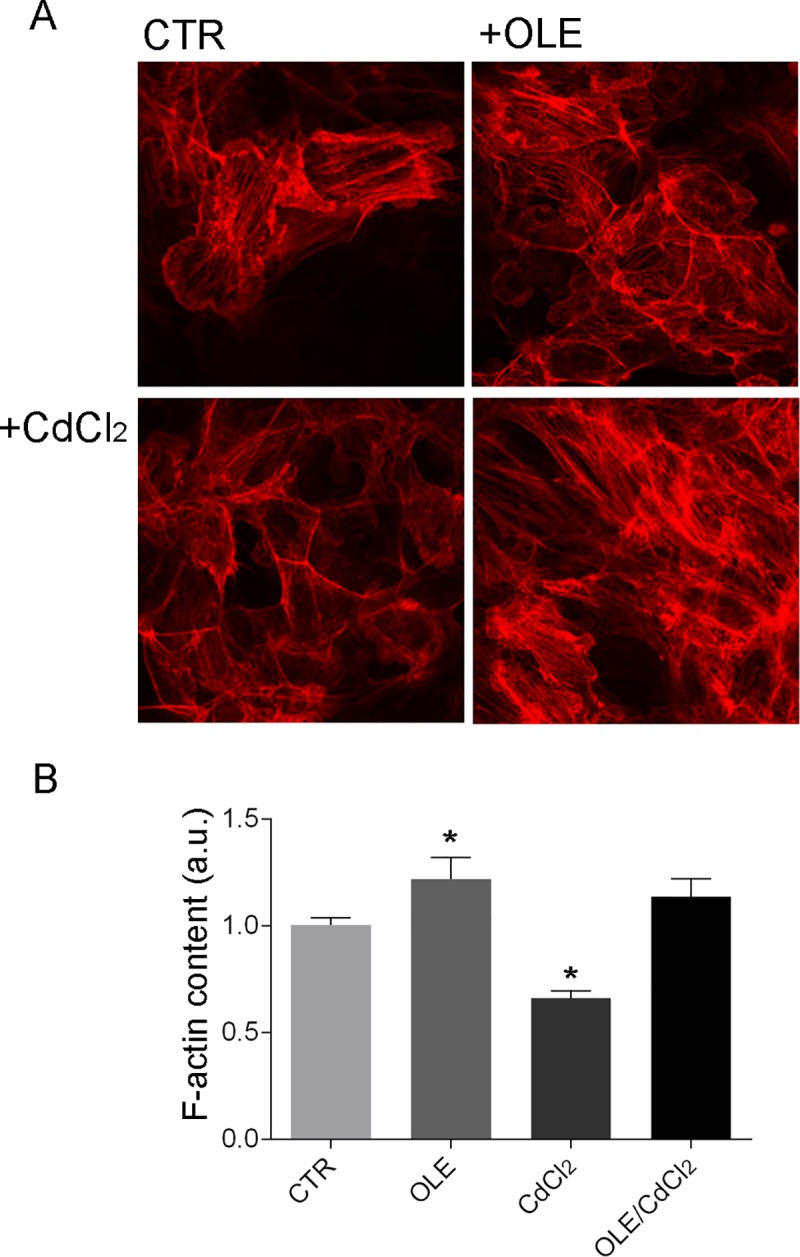
F-actin visualization. (A) Cells were left under basal condition (CTR) or treated with OLE (0.01 mg/ml), CdCl_2_(0.1μM) or with OLE in the presence of CdCl_2_. Specimens were incubated with Phalloidin-TRITC (400 μg/ml) for 45 min at room temperature to detect F-actin. (B) F-actin content was semi-quantified by actin polymerization assay. Confluent cells were treated as described in Methods. After staining with TRITC-phalloidin, cells were extracted with cold methanol and the fluorescence absorbance of extracts was read (540/565 nm) in an RF-5301PC fluorimeter. The values obtained were compared using one-way Anova and Tukey’s multiple comparison test (*P<0,05 vs CTR).

Actin visualization with Phalloidin-TRITC revealed that low cadmium exposure deeply depolymerizes actin filaments compared to cells left under control conditions (Fig 10A). Semi-quantitative analysis of the amount of F-actin detected using the actin polymerization assay (Fig 10B) confirmed that F-actin content significantly decreased on pre-incubation with cadmium. In contrast, incubation with OLE prevented cadmium-induced actin depolymerization.

Interestingly, OLE treatment impairs cadmium induced actin depolymerization. Compared to cells left under basal condition, incubation with the olive leaf extract alone at 0.01mg/ml stabilizes actin filaments (Figs 4–10).

**Fig 11:**
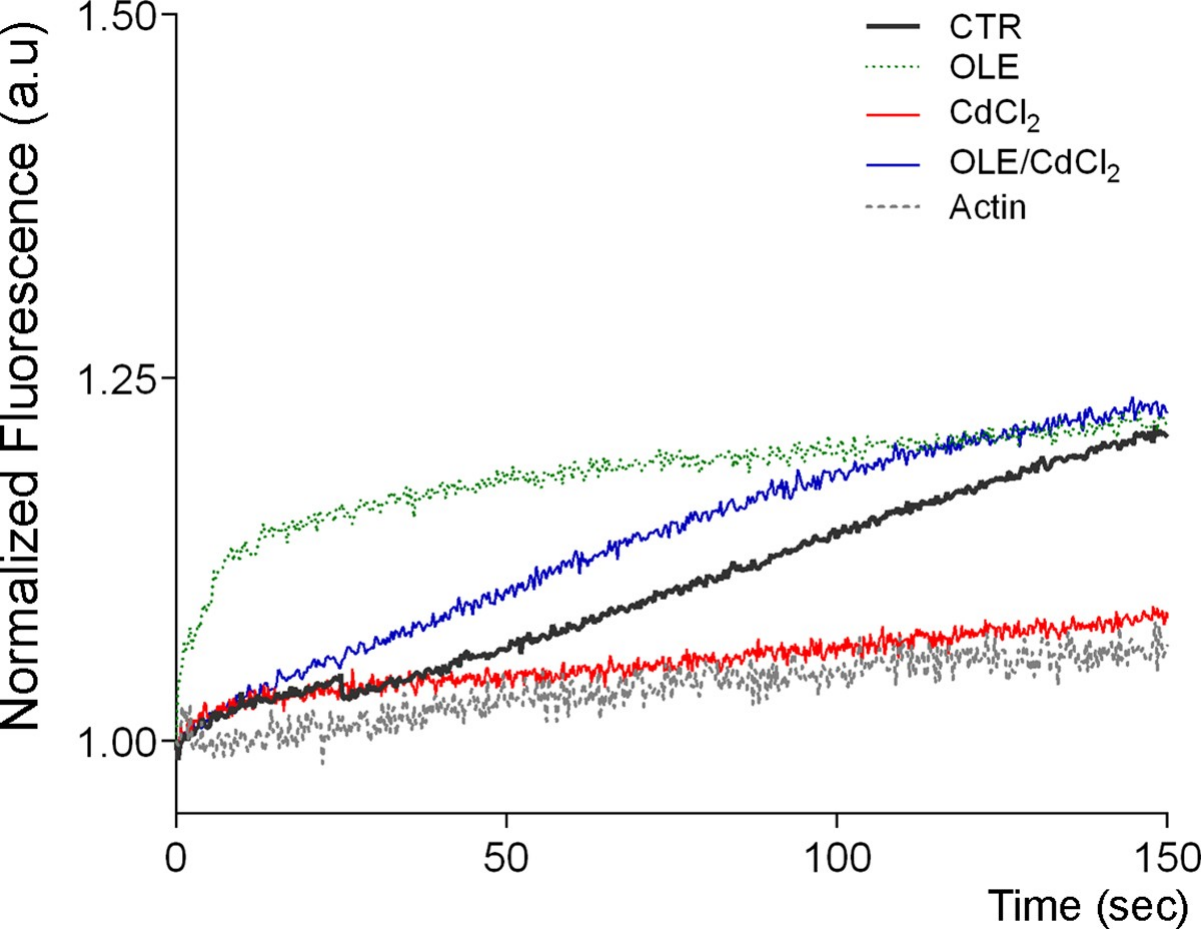
Pyrene-actin polymerization assay. Cytosolic fractions, isolated from cells treated as described above, were incubated with pyrene actin and changes in fluorescence were measured over time. The results shown are representative of three independent experiments performed in triplicate.

Fluorescence-based pyrene-actin assay was performed using cytosolic fractions to further investigate the effect of OLE on actin remodeling (Fig 11).

Polymerization was observed as a time dependent increase in pyrene actin fluorescence as described in Methods. OLE treatment significantly increased the rate of actin polymerization regardless of cadmium treatment. As an internal control, the fluorescence of actin-pyrene was evaluated in the absence of cytosol.

**Fig 12:**
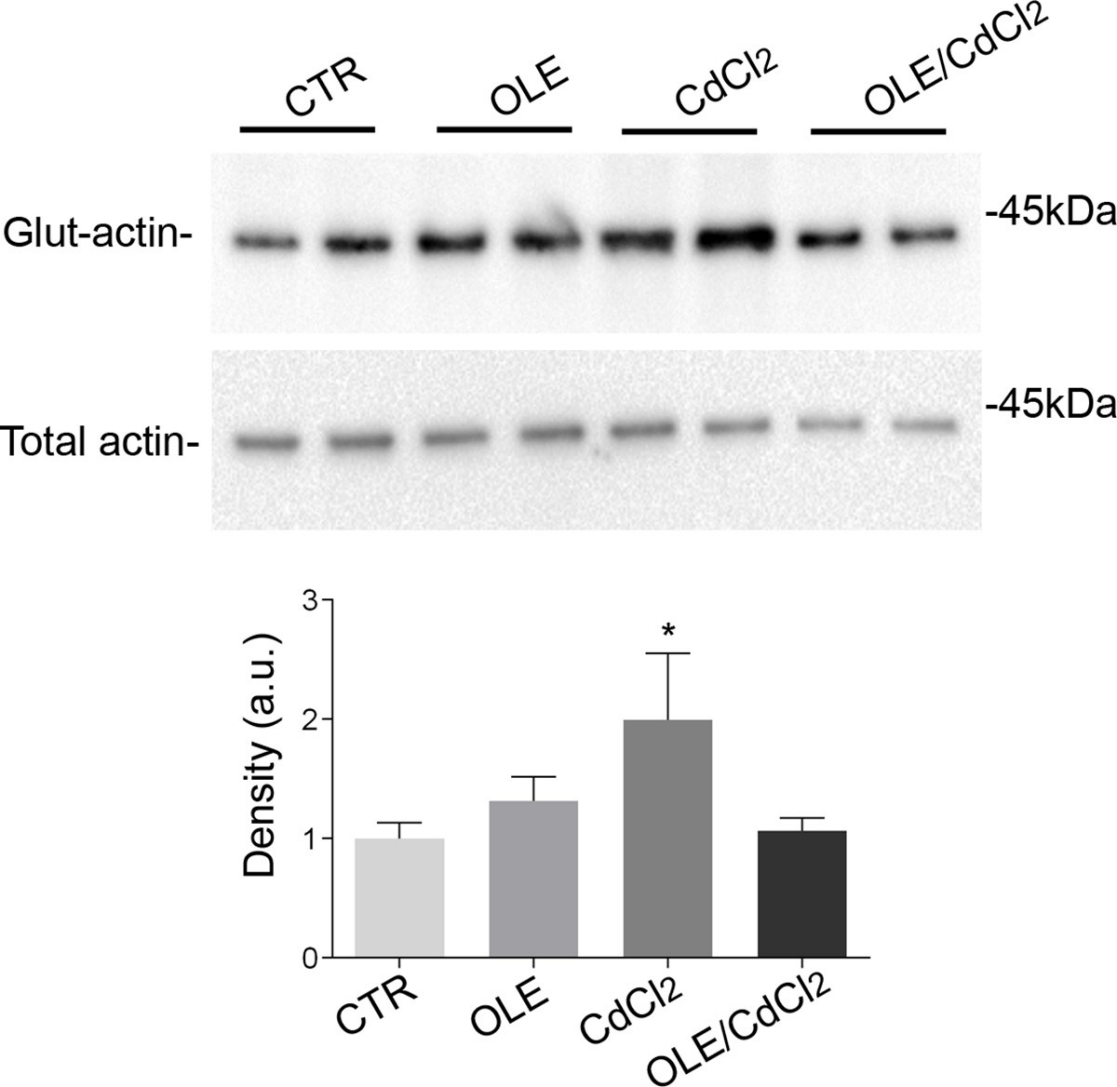
Glutathionylation assay with BIOGEE. Glutathionylated actin was detected as described in Methods. Densitometric analysis of the obtained bands revealed that actin S-glutathionylation significantly increased in cells treated with CdCl_2_ with respect to CTR. The data (means ± SEMs) were analyzed by one-way ANOVA, followed by Tukey’s Multiple Comparison test.

Cadmium exposure induces actin glutathionylation that breaks the dynamic and endogenous intracellular equilibrium between the monomeric G-actin and the filamentous F-actin thereby favoring actin depolymerization [23].Actin S-glutathionylation was analyzed using BioGEE reagent, a cell-permeant biotinylated glutathione ethyl ester developed for the detection of protein glutathionylation. After affinity precipitation of cellular glutathionylated proteins with streptavidin-agarose beads, followed by immunoblotting, the increase of S-glutathionylation of actin induced by cadmium was reversed by co-incubation with OLE (Fig 12; S1 Fig). Treatment with the extract alone did not alter actin S-glutathionylation significantly.

## Discussion

### Cytoprotective effects of olive leaf extracts in renal cells exposed to a low level of cadmium

Olive leaves represent a waste from both olive orchards and the olive oil industry. However, alternative recovery of this biomass may strengthen economic and environmental sustainability. Several studies have been focused on exploiting olive leaves for their potentiality in several fields including pharmacological and food industries. It has been recently shown that olive leaves extracts (OLE) can extend the shelf life of salmon burgers [43] and improve the nutritional value of table olives [44] as well as decrease lipid oxidation in baked snacks [3]. Furthermore, OLE may have beneficial effects on human health [5,12,45,46].

Most of the biological actions of OLE are related to its high content of polyphenols, even if the synergic contribution of other components in the extract cannot be excluded.OLE administration has been shown to reduce cell proliferation and cyst growth in a cell model of autosomal dominant polycystic kidney disease [47]. OLE can scavenge superoxide thereby inhibiting the hemolysis process in human erythrocytes [46]. In vivo studies have revealed that aqueous and ethanol extracts obtained from olive leaves exert antioxidant effects and have hypocholesterolaemic proprieties [48] as well as being able to modify the immune response by increasing IFN-γ production and the levels of NO, suggesting a potential cardio-protective action [49].Previous findings from our group have demonstrated that olive leaf extracts obtained from the local *Coratina* cultivar significantly reduce tBHP-induced ROS generation in bronchiolar NCI-H292 cells [16]. Here, the efficacy and the physiological effects of OLE have been investigated in renal collecting duct MCD4 cells exposed to a low cadmium level. Cadmium is a high toxic heavy metal and human exposure to cadmium has dramatically increased over the years. The major route of human exposure to cadmium is through inhalation and oral ingestion as cadmium accumulates in leaves of leafy vegetables [50]. Another relevant source of chronic exposure to cadmium is tobacco smoking [51]. Several epidemiological studies have revealed that chronic exposure to low cadmium levels, occurring in industrialized countries, increases the risk of health damage [52–54]. Therefore, the effects of low doses of cadmium were investigated. Following exposure, cadmium accumulates in several organs including the kidneys. Urinary cadmium is actually considered an environmental exposure biomarker [55].In workers occupationally exposed to cadmium, urinary cadmium concentration higher than 5 μg/g is significantly associated with oxidative stress [51].

Renal tubular dysfunctions have been described following long-term low-level exposure to cadmium [56].In the kidney, TRPV5 and TRPV6 are the major routes for cadmium entry into the cells, though they mainly mediate calcium transport in distal convoluted and connecting renal tubules [57].TRPV6 channels are endogenously expressed in MCD4 cells [58], where they might constitute a possible pathway for cadmium entry. Once inside the cells, cadmium severely affects cell physiology and growth. Most effects associated with cadmium exposure result from its pro-oxidant proprieties. Therefore, agents displaying antioxidant abilities may be promising to prevent or reduce cadmium induced oxidative stress and its associated harmful consequences at the cellular and molecular level.

Several plant extracts including OLE can modulate cell behavior and counteract the environmental toxicity exposure [59]. At the concentrations used here, OLE treatments do not affect cell viability as assessed by crystal violet measurement. Conversely, OLE counteracts several toxic actions of cadmium possibly by reducing ROS generation. Indeed, cadmium exposure causes DNA damage and chromosomal injury possibly by inducing oxidative stress [60]. In this respect, we found that treatment with OLE significantly reduces cadmium-induced ROS production. Micronuclei formation is often used as a general biomarker to test cell exposure to genotoxic pollutants such as heavy metals. Interestingly, our treatments with OLE abolished cadmium induced micronuclei formation. Comet assay was further applied as an additional exposure biomarker showing that OLE contrasts cadmium action by ameliorating several comet parameters.

Exposure to cadmium reduces cellular defense by inactivating antioxidant enzymes. Under this condition, the intracellular content of reactive species increases and leads to oxidative stress that may cause several cellular dysfunctions including lipid accumulation [61]. Lipid droplets are intracellular organelles described in almost all cells under physiological and pathological conditions. The intracellular abundance of lipid droplets can increase dramatically upon exposure to xenobiotics and heavy metals such as cadmium [39,42]. Detection of lipid droplets is achieved in fixed cells using oil-red-O staining [42].A slight but not significant increase in lipid droplets was detected in cells incubated with OLE alone. In addition to polyphenols, OLE contains several metabolic substrates that may promote lipid droplet formation as an intracellular lipid storage site [42]. In contrast, low cadmium exposure significantly promotes the formation of lipid droplets, possibly as a compensative and protective response in stressed cells. Interestingly, we also found that OLE prevents the formation of lipid droplets induced by low cadmium exposure, though the molecular mechanism remains to be established.

### Olive leaf extract modulates actin remodeling in renal cells exposed to low cadmium

The tight modulation of actin dynamics is fundamental for numerous cellular functions such as cell adhesion, migration and intracellular trafficking. In rat mesangial cells, cadmium causes actin depolymerization, affecting the functionality of the glomerulus and deregulating the glomerular filtration rate [23]. Renal collecting duct cells play a pivotal role in controlling water and salt homeostasis. Structural cell integrity is therefore required for kidney tubular functions. Several factors and signals have been shown to be involved in controlling actin polymerization and depolymerization dynamics [62]. In the literature it has been reported that ROS negatively modulate actin polymerization [63]. In this study, treatment with the oxidant tBHP promotes ROS production and severely disrupts actin filaments. Interestingly, the negative action of tBHP on actin depolymerization was restored by pre-incubation with OLE. Fluorescence-based pyrene-actin assay revealed that the rate of actin polymerization was significantly increased by OLE and reduced by cadmium as already shown in other cell models [22]. Interestingly, incubation with OLE attenuates the negative action of cadmium on actin remodeling. Moreover, ROS may promote actin depolymerization favoring its S-glutathionylation [63]. In this respect, it is well established that low cadmium administration increases actin S-glutathionylation [23]. In the present study, we demonstrate that OLE reduces cadmium-induced actin S-glutathionylation thereby stabilizing actin filament, possibly by decreasing ROS generation. These findings reveal for the first time that OLE can modulate actin remodeling by affecting S-glutathionylation processes.

## Conclusions

**Fig 13:**
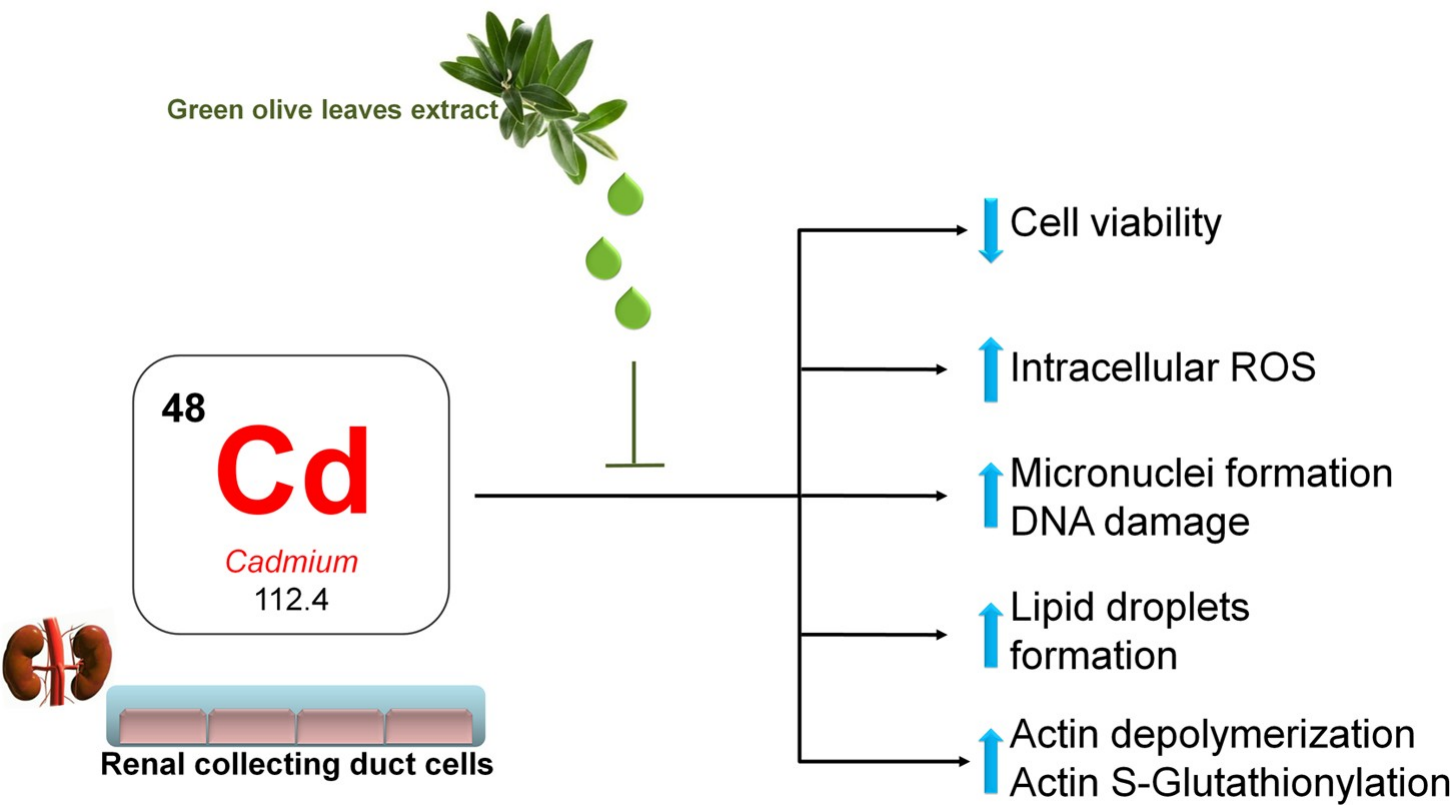
Proposed model of the action of olive leaf extract in renal cells exposed to a low cadmium dose. Details in the discussion.

Low cadmium exposure causes cell injury, possibly through inducing ROS production. The present findings reveal that treatment with OLE antagonizes the adverse effects of cadmium and decreases cadmium induced ROS generation in renal cells. A proposed model of cadmium action in renal cells is shown in Fig 13.

These observations provide a novel insight into the biological activity of OLE in renal collecting duct cells and indicate that OLE may represent a potential adjuvant against chronic low cadmium exposure.

Administration of OLE as an antioxidant agent would be, indeed, promising in preventing cadmium toxicity in environmentally and occupationally exposed populations or in habitual tobacco smokers who are chronically exposed to cadmium. Furthermore, olive leaves, considered waste from olive groves and the olive oil industry, might be potentially applied in bioremediation programs. The present data suggest the potential of exploiting olive leaf biomass, which displays several beneficial biological actions, in promoting economic and environmental sustainability.

## Supporting information

S1 Fig Original blots and membranes of Fig 12. The upper blot shows actin glutathionylation. In contrast the blot below indicates the total abundance of actin in the lysates. Obtained bands in the blots were normalized to total protein using the stain-free technology (Bio-Rad, Segrate Milano Italy).
(TIF)
