# Supplementary figures and images for: Green olive leaf extract (OLE) provides cytoprotection in renal cells exposed to low doses of cadmium
Source: PLoS One. 2019 Mar 21;14(3):e0214159. doi: 10.1371/journal.pone.0214159 (PMC6428325; doi:10.1371/journal.pone.0214159)

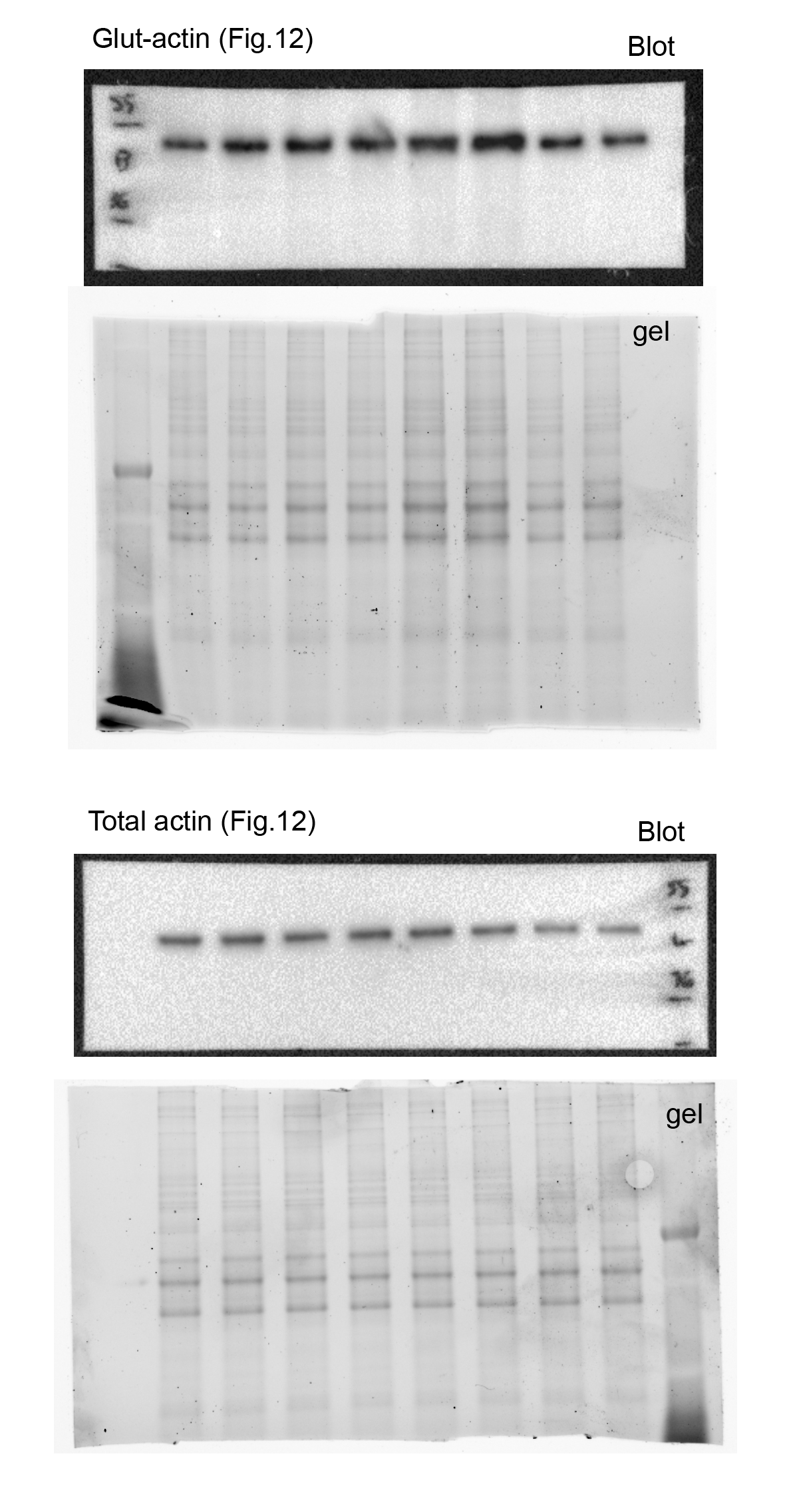

Supplement: S1 Fig — The upper blot shows actin glutathionylation. In contrast the blot below indicates the total abundance of actin in the lysates. Obtained bands in the blots were normalized to total protein using the stain-free technology (Bio-Rad, Segrate Milano Italy). (TIF) [file pone.0214159.s001.tif]
